# An Alternative Site for Pin Placement in External Fixation of Pelvic Fractures: Lateral Posterior Pelvic External Fixator Surgical Technique

**DOI:** 10.3389/fsurg.2020.621125

**Published:** 2021-01-28

**Authors:** Matthias K. Russ, Pierre Navarre, Jarrad P. Stevens

**Affiliations:** ^1^Department of Orthopaedic Surgery, Alfred Hospital, Melbourne, VIC, Australia; ^2^Cabrini Hospital, Orthopaedic Surgery, Melbourne, VIC, Australia; ^3^Department of Surgery, Monash University, Melbourne, VIC, Australia; ^4^Department of Orthopaedic Surgery, Southland Hospital, Invercargill, New Zealand; ^5^Department of Surgical Sciences, University of Otago, Dunedin, New Zealand; ^6^Knox Orthopaedic Group, Knox Private Hospital, Melbourne, VIC, Australia

**Keywords:** pelvic fracture, polytrauma, external fixation, damage-control orthopedics, lateral posterior external fixator

## Abstract

**Introduction:** The application of an external fixator for unstable pelvic fractures is an important component of many resuscitation protocols. Moreover, certain pelvic fractures may be treated with an external fixator without requiring further internal fixation. We report our initial clinical results with an alternate pelvic external fixator site, the lateral posterior external fixator (LPEF), and describe the surgical technique.

**Methods and Materials:** From 2010 to 2013, we identified 27 consecutive patients (mean age 44.6 years, range 18–80 years) treated by the same surgeon (MKR) with an LPEF in a level 1 trauma center. Retrospective data collection included mechanism of injury, surgical interventions, and complications.

**Results:** The LPEF was used in 16 patients as acute pelvic stabilization and converted at a median of 2 days (interquartile range 1–3.5) to internal fixation, whereas in 10 patients, it was used as definitive treatment and removed at a median of 48 days (interquartile range 37–64). One patient died on day 14, secondary to his severe closed head injury. The only surgical complications were two wound infections (20%, 2/10 in the group of definitive LPEFs), which resolved without sequelae after the removal of the LPEF (at 36 and 50 days) and antibiotics, one case of loss of fixation leading to the removal of the LPEF at 71 days, and one patient who had hypergranulating external fixator sites and eventually healed without any cutaneous sequelae. All fractures consolidated in a good position.

**Discussion:** The described techniques of pelvic external fixation include the anterosuperior (iliac wing), supra-acetabular (anteroinferior), and subcristal (anterior superior iliac spine) insertion sites. The reported infection rates in definitive pelvic fracture treatment range from 20 to 40%. Due to the localization of the insertion sites, the lateral femoral cutaneous nerve is potentially at risk with the last two techniques. On the other hand, the LPEF insertion site is quite safe, as it is anatomically far from any nerves and the inguinal region, and allows easy access for laparotomy. The results in this series suggest that the lateral posterior pelvic external fixator technique is an alternative to previous techniques with a low risk of complications.

## Introduction

Application of external fixation for major pelvic trauma is an important component of many resuscitation protocols, with the goal of stabilizing the pelvis to control hemorrhage, decrease blood transfusions rates, and improve survival rates (1–5). The procedure should be simple, quick, have minimal risk of complications, and be reproducible for surgeons who may rarely require its use in regional settings. Moreover, certain pelvic fractures may be treated with an external fixator without requiring further internal fixation.

**Figure 1:**
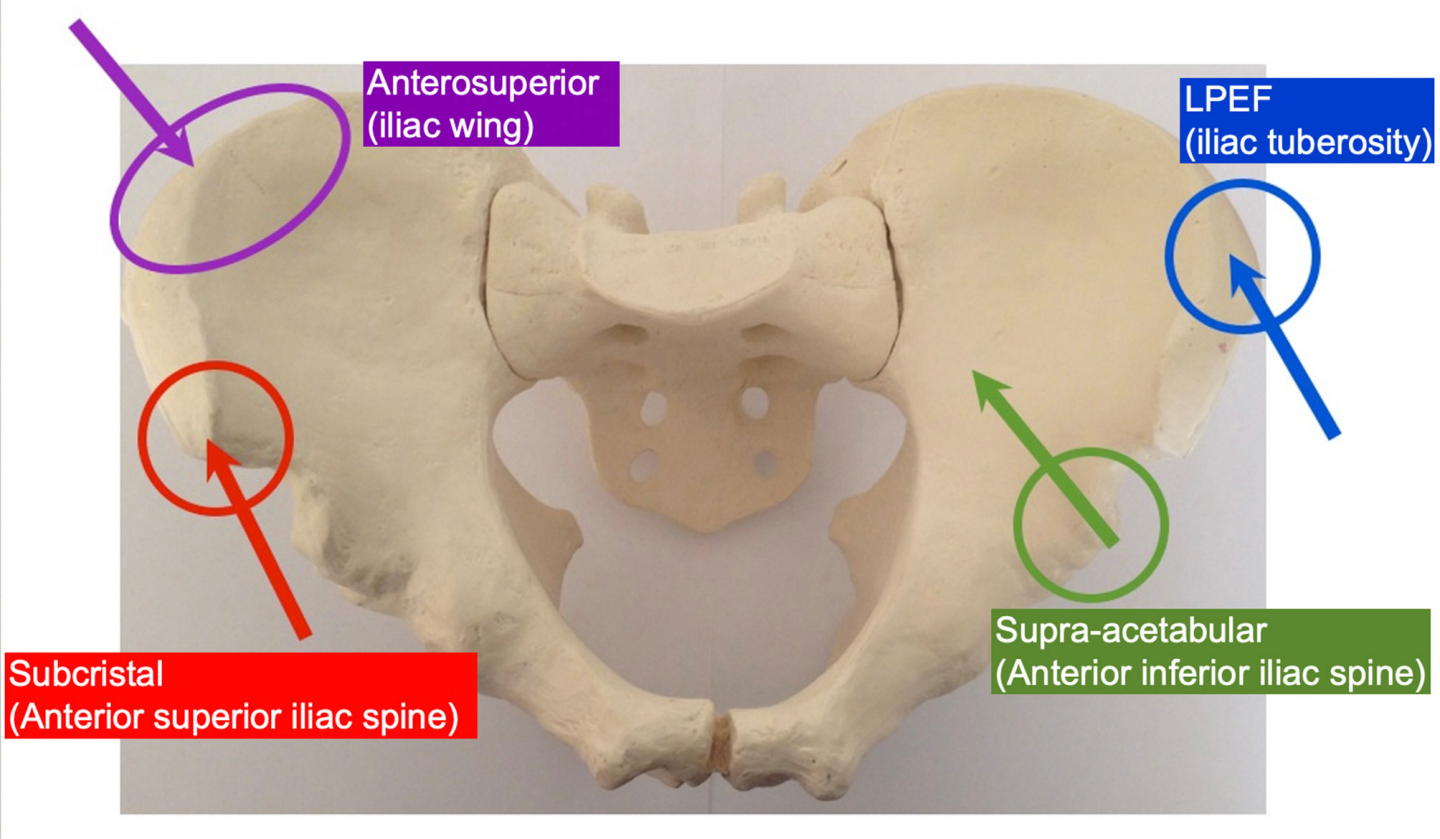
Comparison of different external fixation techniques. *Purple*, anterosuperior (iliac wing). *Green*, supra-acetabular (anterior inferior iliac spine). *Red*, subcristal (anterior superior iliac spine). *Blue*, LPEF, lateral posterior pelvic external fixator (iliac tuberosity).

The surgical anatomy of three techniques for anterior pelvic external fixation has been well-described, including anterosuperior (iliac wing), supra-acetabular or anteroinferior (anterior inferior iliac spine), and subcristal [anterior superior iliac spine (ASIS)] insertion sites (6). Associated risks of complications are well-recognized, including pin malposition, loss of fixation, neurovascular injury, and infection (6–10). We describe the initial clinical results using a lateral posterior pin site entry technique, which we have named the lateral posterior external fixator (LPEF). Using the tuberous portion of the lateral iliac crest as an identifiable landmark, we can generate a lateral entry point and directing posteriorly, thus positioning our pins further away from the groin crease and skin folds caused by the sitting position (Figure 1). The senior author (MKR) has developed and used this technique since 2010, and it is currently his technique of choice in many pelvic fracture configurations, both for initial stabilization and definitive treatment.

We have received approval from the Alfred Hospital Ethics Committee (466/13) for this study.

## Methods and Materials

We have identified 27 consecutive patients who have been treated with an LPEF by a single surgeon (MKR) between 2010 and 2013. The patients were identified by index codes from medical records at The Alfred, a level 1 referral trauma center in Victoria, Australia. Inclusion criteria were patients older than 18 years of age treated with an LPEF for acute treatment of a pelvic ring injury. Exclusion criteria were patients who had a different type of pelvic external fixator, other than the LPEF. We proceeded to a retrospective medical records review and collected data relative to injury mechanism, surgical interventions, complications, including infection (superficial pin site infection defined as cellulitis and purulent discharge responding to dressings and pin site care or removal of external fixator without evidence of osteomyelitis, deep infection defined as osteomyelitis, or deep collection), cutaneous complications (such as hypergranulating wounds), iatrogenic fracture, gross malposition of the external fixator (missing the iliac tuberosity on postoperative radiographs), loss of fixation, nerve or vascular injury, and demographic data. Lateral femoral cutaneous nerve injury was defined by clinical examination of numbness or loss of sensation in the anterolateral proximal thigh. Descriptive statistics for all sociodemographic variables and injury factors will be reported using mean and range for normally distributed continuous data, mean and interquartile range (IQR) for continuous data that were not normally distributed, and proportions for categorical variables. All analyses were performed using Stata Version 13.

### Surgical Technique

The patient is positioned supine on a radiolucent table, our preference being the Jackson table. This allows easy application of the pelvic external fixator, external or internal fixation of any fractured limbs, and access for the image intensifier. Draping of the patient should allow access to the entire abdomen down to the level of the table and the proximal thighs, excluding the genital area from the sterile field. This allows access for laparotomy as required and access for added external fixator sites as required. If there are fractures of the lower limbs, they can be included in the sterile field in the same setting to minimize operative time.

**Figure 2:**
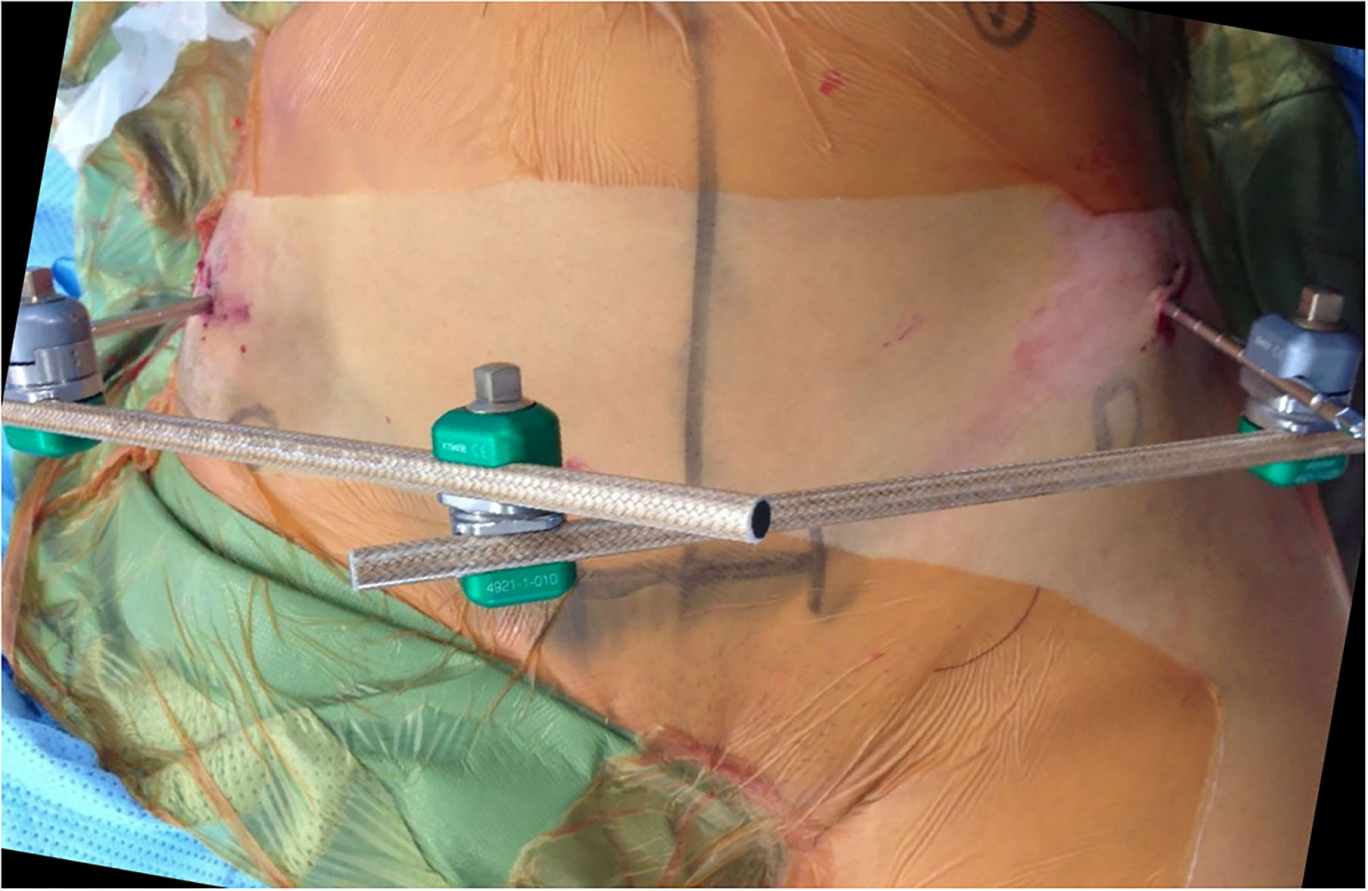
LPEF technique, view from above. Pin insertion posterolateral to the ASIS (marked by circles) in slight lateral to medial and caudal to cranial direction.

**Figure 3:**
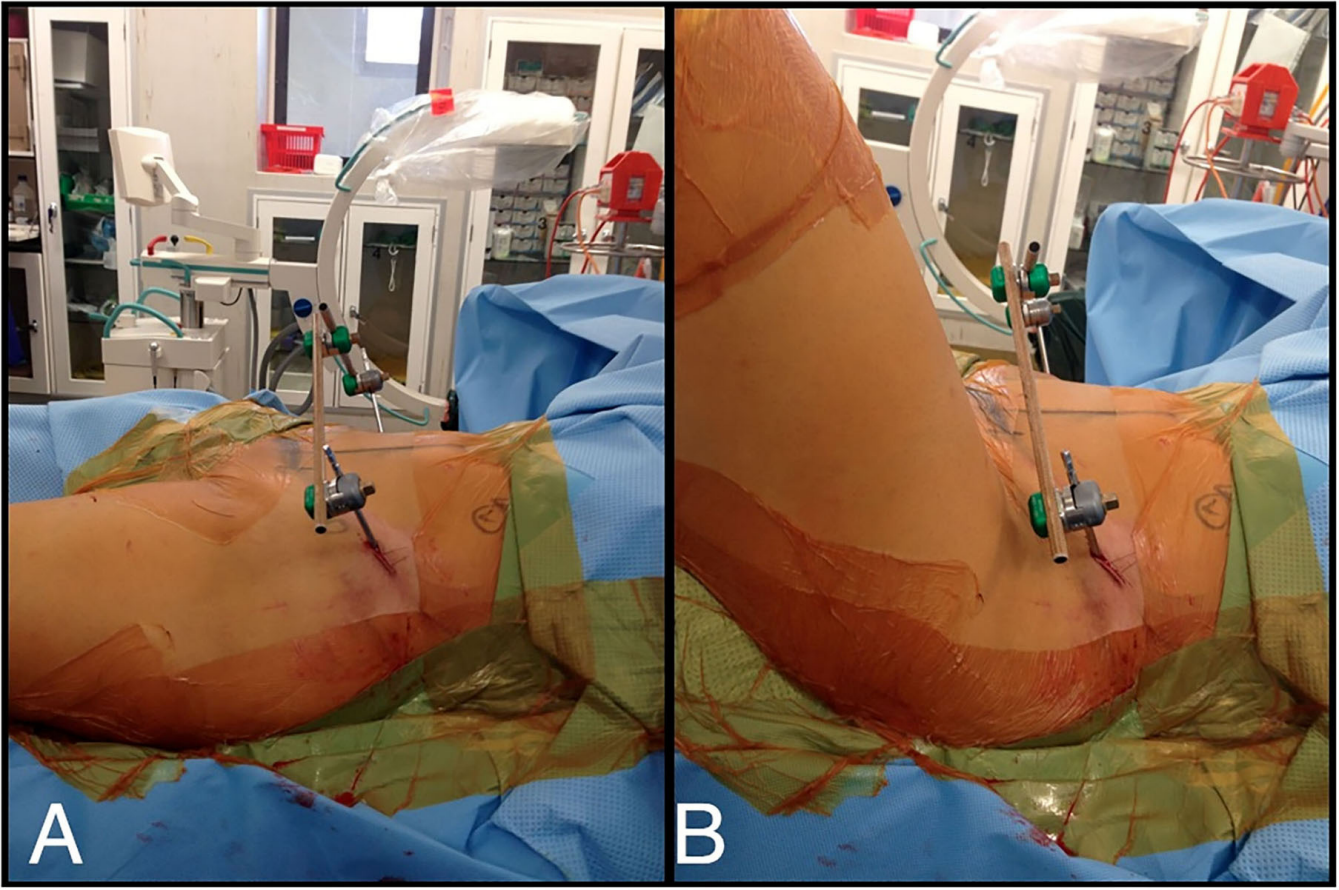
**(A)** LPEF technique, view from side. **(B)** Flexion of the hips not hindered by LPEF pins and flexion creases far from LPEF insertion site.

**Figure 4:**
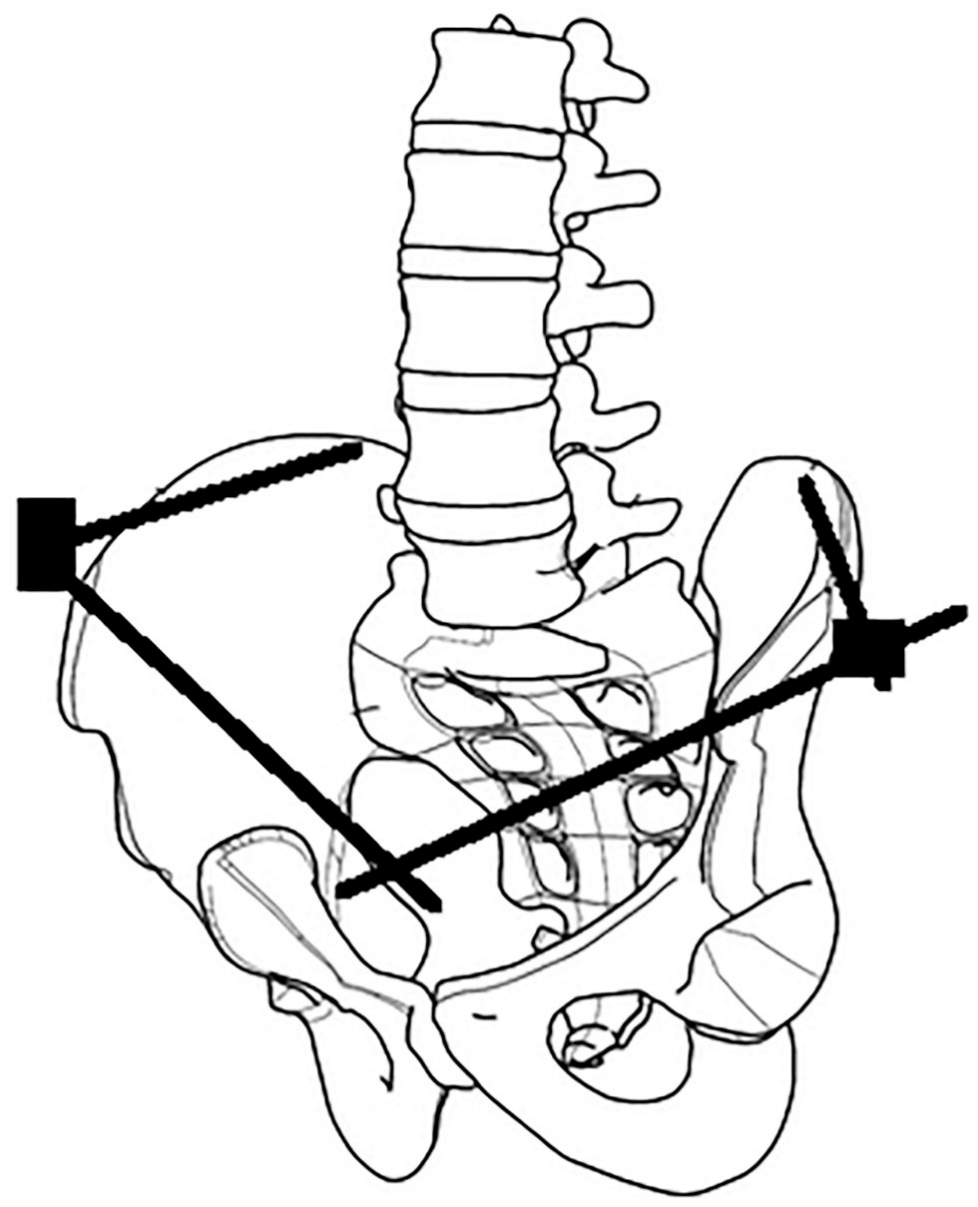
Line-drawing image representing the position of the LPEF.

**Figure 5:**
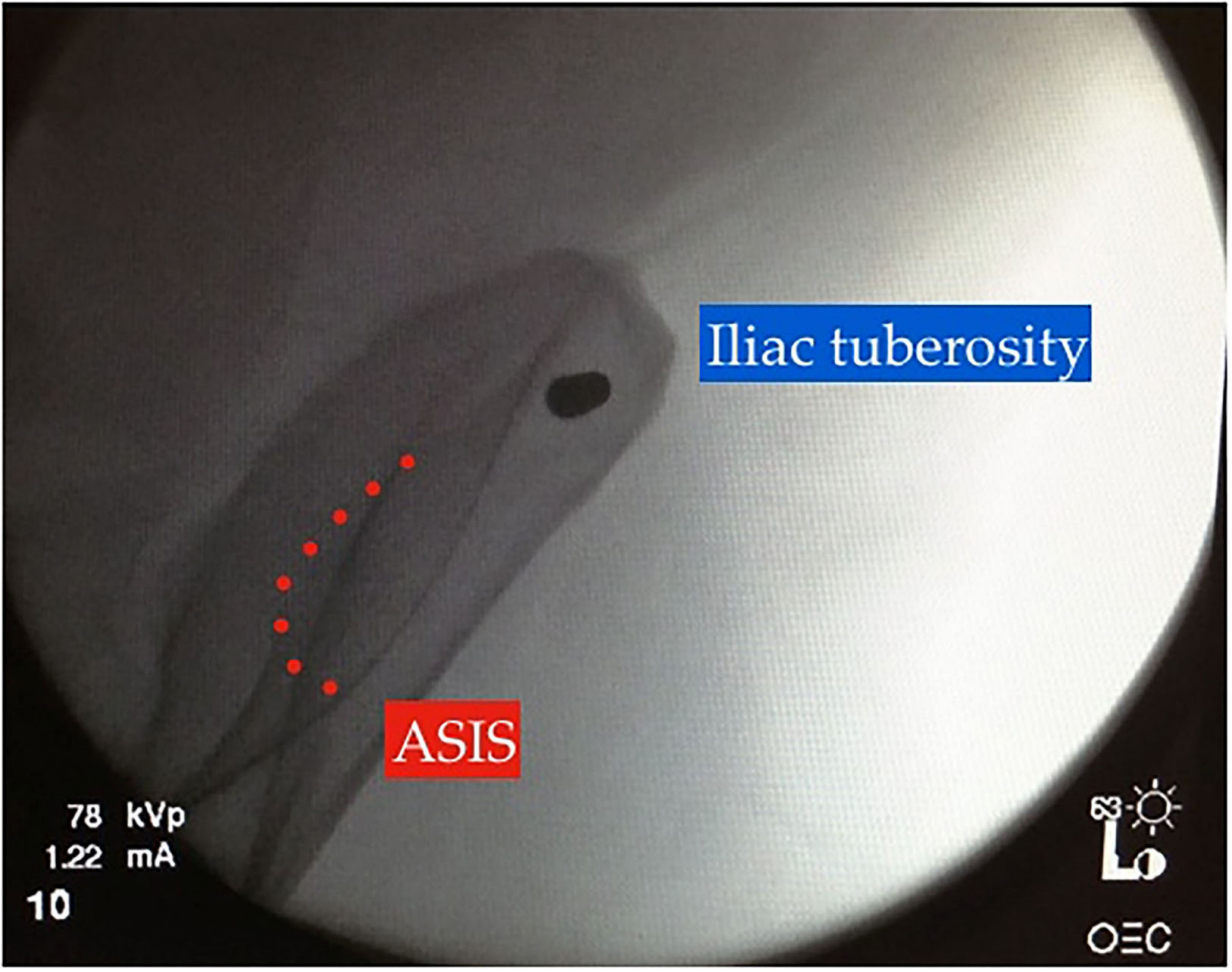
“Bull's eye” view on intraoperative image intensification, modified obturator-oblique view confirming adequate pin position within the iliac tuberosity.

**Figure 6:**
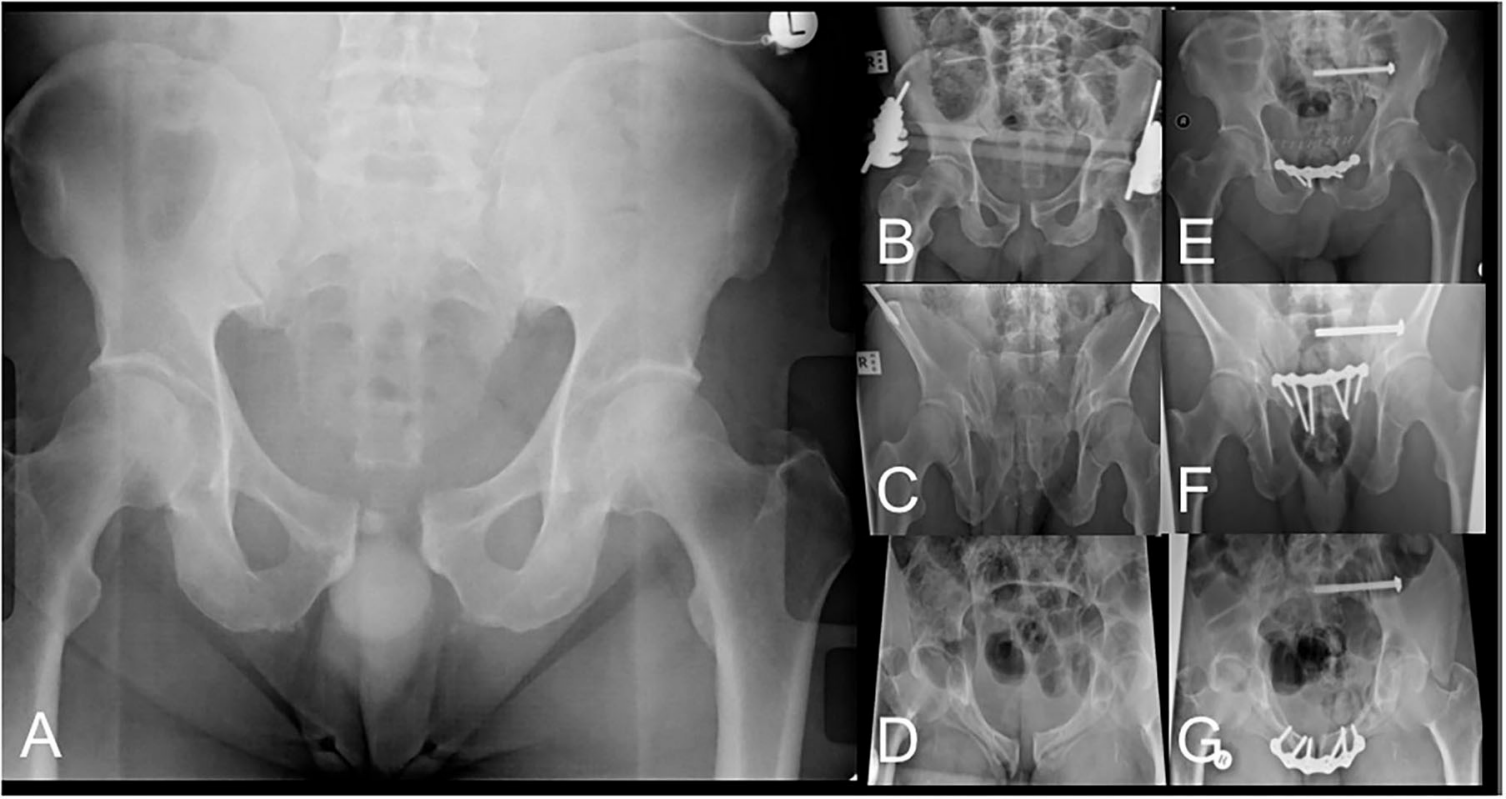
AP pelvis radiograph **(A)** of pelvic injury classified as left-sided APC2. AP **(B)**, outlet **(C)** and inlet **(D)** radiographs after LPEF application demonstrating improved pelvic alignment awaiting definitive surgical management. AP **(E)**, outlet **(F)** and inlet **(G)** radiographs after definitive fixation after removal of LPEF.

Identifiable landmarks include the ASIS and the iliac crest, which is palpated posterolaterally from the ASIS. The tuberous portion of the iliac crest is palpated as the iliac crest curves posteriorly and medially. These landmarks are outlined with a surgical marking pen (Figure 2). A 2-cm incision is made overlying the tuberous portion of the iliac crest in the superolateral to inferomedial direction, allowing incision extension as required. An artery clip is used to bluntly dissect the subcutaneous tissues and reach the tuberous portion of the iliac crest. This is a percutaneous technique; however, two small retractors can aid in visualization as required. The periosteum is carefully incised in its center with a 15-blade scalpel or electrocautery. A 5-mm by 150 or 180-mm partially threaded self-drilling and a self-tapping pin is used with a soft-tissue protection sleeve carefully inserted to the pin entry point. The orientation is from anterior to posterior in a slight caudal to cranial direction and from lateral to medial following the direction identified by palpation of the iliac crest with the surgeon's free hand, usually at ~20°. An artery clip or the blunt aspect of a K-wire can also be inserted on the lateral inferior aspect of the iliac wing to guide the alignment of the pin. The pin is advanced at low-speed to remain between the medial and lateral cortices as long as possible and can be either uni-cortical or bi-cortical depending on the working length achieved (Figures 2–4). Of note, we have found it easier to put the pins in while standing on the contralateral side of the patient. Image intensifier can be used to guide the direction of the pin; however, we have been very successful in confirming good positioning after both pins have been inserted. A standard AP view of the pelvis is obtained, as well as a modified obturator oblique-outlet view performed in a way to center the pin in a “bull's eye” fashion, often rotating ~20° from the AP view toward the obturator oblique view, and adding ~10°s of outlet view until the pin is centered on the imaging (Figure 5), which confirms that the pin is central in the tuberous region of the iliac bone. The external fixator construct is completed with 2 or 3 carbon-fiber rods connected with pin-to-rod clamps and rod-to-rod clamps, and the pelvis is reduced with a combination of external manipulation or traction, often using the pins as “joysticks.” The construct is then tightened. The image intensifier is used with standard AP, inlet and outlet views to assess the quality of the reduction of the anterior and posterior pelvic ring (Figure 6). Procedural time is in the order of 10 min, similar to the subcristal technique (8).

**Figure 7:**
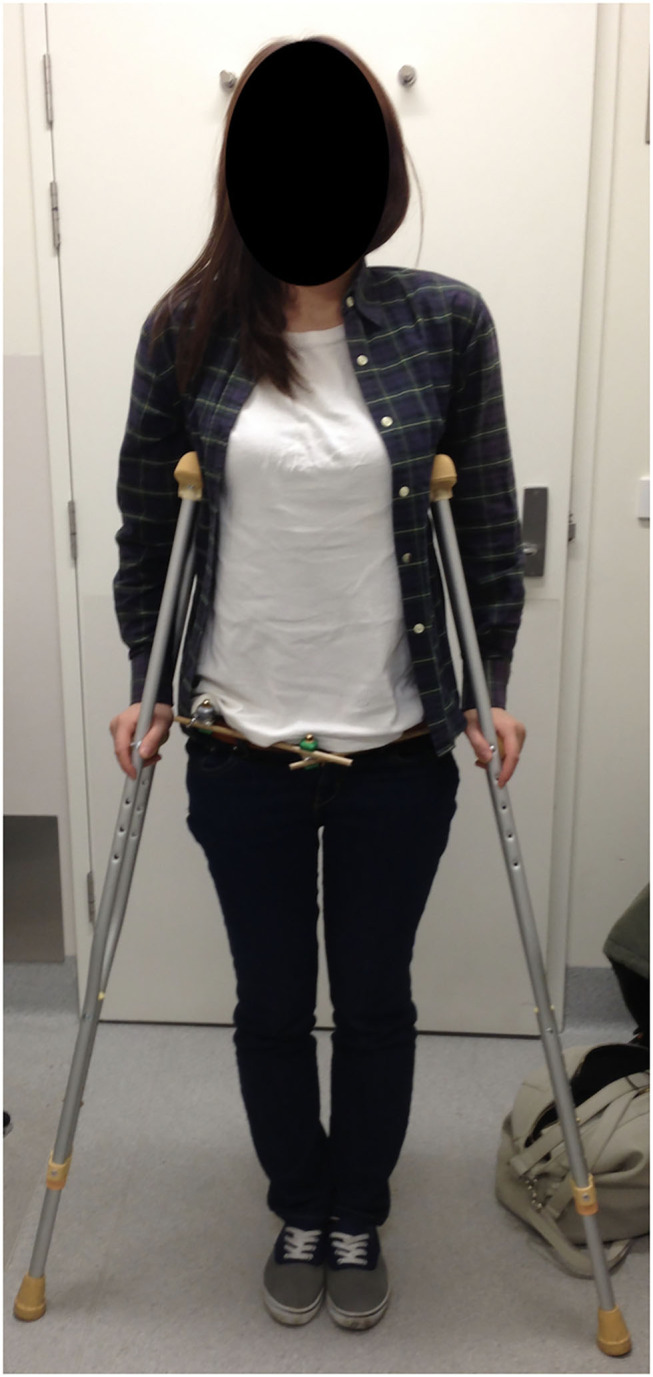
Patient at 2 weeks after LPEF demonstrating ease of clothing and mobility. Weight-bearing dictated by fracture configuration, this specific patient had a left-sided LC1 injury and was allowed full weight-bearing on the right and protected on the left with crutches.

The postoperative care includes regular dry-dressings of the pin sites and observation for any signs of pin site infection. Weight-bearing status is dictated by fracture configuration (Figure 7). Follow-up in the clinic with radiographs occurred at 2, 6, and 12 weeks and 6 months at least until clinical and radiological union with healed surgical wounds or death.

## Results

**Figure 8:**
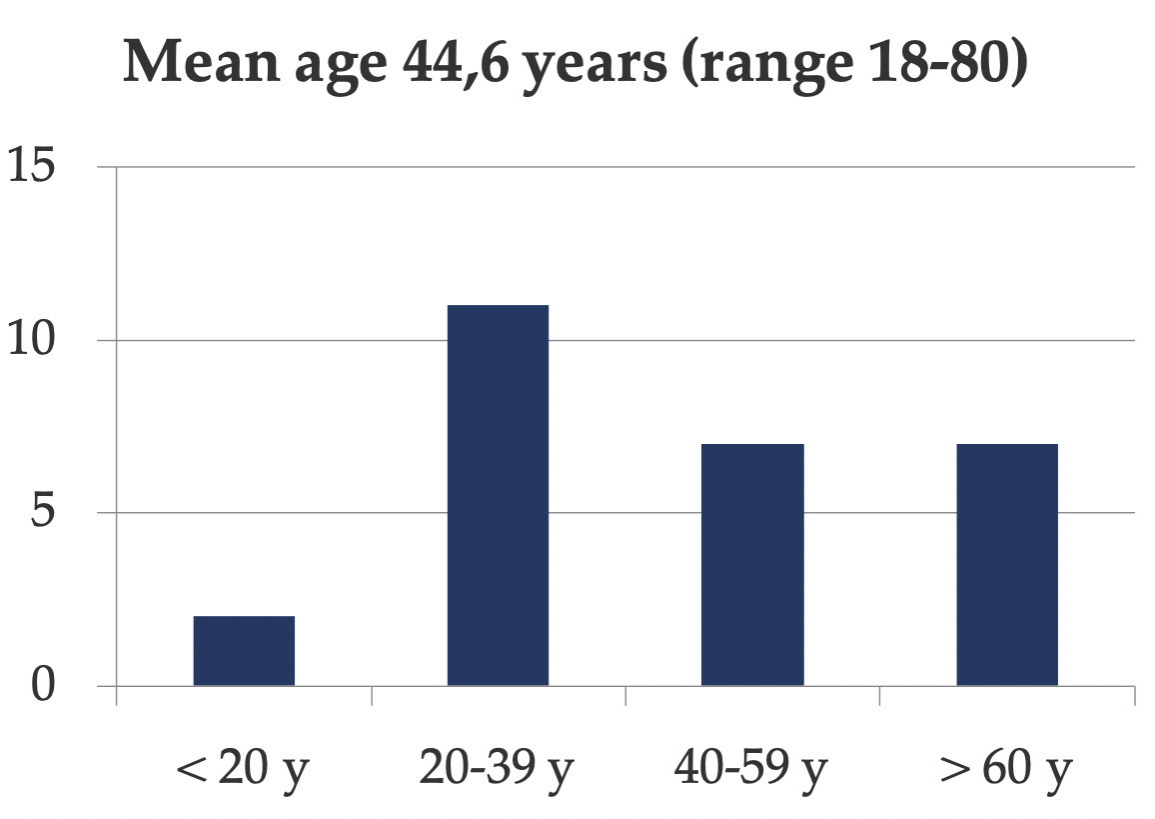
Histogram demonstrating that the LPEF was used in patients of all age categories.

The cohort of 27 patients included 17 males and 10 females with a mean age of 44.6 years (range 18–80 years) and distributed among all age categories (Figure 8). Demographic data and results are summarized in Table 1. The external fixators have been used as an acute stabilization procedure in 16 patients and as a short-term “definitive” fixation in 10 patients. One patient died on day 14 from the sequelae of his severe traumatic brain injury. In the temporary external fixator group, the external fixator was a temporary stabilization method in the hospital's resuscitation algorithm in patients with unstable pelvic fractures and ongoing hemodynamic instability. These external fixators were then removed during definitive pelvic fixation, at a median time of 2 days postoperatively (IQR 1–3.5). In the latter group, they were removed at a median time of 48 days postoperatively (IQR 37–64), when the fractures were considered healed enough by radiological and clinical criteria for external fixation removal.

**Table 1:** Patients' demographic data and results.

**Patient**	**Age at injury**	**Gender**	**Y-B classification**	**Mechanism**	**Transfer**	**Days of hospitalization**	**Days of definitive LPEF**	**Days of temporary LPEF**	**Converted to**	**Laparotomy**	**Thoracotomy**	**Infection**	**Skin problems**	**Fracture**	**Malposition**	**Loosening**	**Nerve injury**
D1	34	M	LC3	MVA	Direct	23	71			Yes	No	No	Hypergranulation ex-fix sites	No	No	Yes but function	No
D2	31	F	LC2	MVA	Direct	31	67			No	No	No	No	No	No	No	No
D3	61	F	LC3	Crush	Transfer	17	64			No	No	No	No	No	No	No	No
D4	31	F	LC3	Pedestrian-car	Transfer	10	52		LPEF + posterior pelvis fixation	Yes	No	No	No	No	No	No	No
D5	70	M	LC3	Crush	Direct	7	50		LPEF + R + L SI screws	No	No	Yes—required removal ex-fix at 50 days post-op	No	No	No	No	No
D6	44	F	LC1	MVA	Direct	10	46			No	No	No	No	No	No	No	No
D7	20	M	LC3	Pedestrian-truck	Direct	58	45			Yes	No	No	No	No	No	No	No
D8	72	F	LC3	Pedestrian-car	Direct	39	37			No	No	No	No	No	No	No	No
D9	18	F	LC3	Fall horse	Direct	6	36			No	No	Yes—required removal ex-fix at 36 days post-op	No	No	No	No	No
D10	80	F	LC3	Pedestrian-tractor	Direct	10	36			No	No	No	No	No	No	No	No
T1	58	M	LC2	MBA	Transfer	5		11	Posterior pelvis fixation	No	No	No	No	No	No	No	No
T2	68	M	APC3	Fall height	Direct	10		5	Anterior ORIF Pfennesteil + L SI screw	No	No	No	No	No	No	No	No
T3	63	M	VS R comb with R acetabulum	Fall horse	Transfer	33		4	Anterior ORIF (bilat Stoppa) + 2 R SI screws	Yes	No	No	No	No	No	No	No
T4	25	M	APC2	MBA	Direct	19		4	Anterior ORIF Pfennesteil + posterior pelvis fixation	No	No	No	No	No	No	No	No
T5	44	M	LC3	MBA	Direct	10		3	Anterior ORIF (bilat Stoppa)	No	No	No	No	No	No	No	No
T6	56	M	LC3	MBA	Direct	18		3	Anterior ORIF Pfennesteil + posterior pelvis fixation	No	No	No	No	No	No	No	No
T7	34	M	APC2	MBA	Direct	6		3	Anterior ORIF Pfennesteil + R SI screw	No	No	No	No	No	No	No	No
T8	61	F	LC2	Pedestrian-truck	Direct	19		2	Posterior pelvis fixation	Yes	No	No	No	No	No	No	No
T9	30	F	LC3 comb with L acetabulum	Fall height	Direct	35		2	Ant plus SIJ plus post column + other	Yes	No	No	No	No	No	No	No
T10	36	M	VS	MBA	Direct	15		1	Anterior ORIF (bilat Stoppa) + L SI screw	No	No	No	No	No	No	No	No
T11	33	M	VS R comb with L acetabulum	MVA	Direct	39		1	Anterior ORIF (bilat Stoppa) + posterior pelvis fixation	No	No	No	No	No	No	No	No
T12	52	M	APC2	MBA	Direct	16		1	Anterior ORIF Pfennesteil	No	No	No	No	No	No	No	No
T13	55	M	APC2	Crush	Transfer	25		1	Anterior ORIF Pfennesteil + R SI screw	Yes	Yes	No	No	No	No	No	No
T14	21	F	LC3	Pedestrian-car	Direct	9		1	Anterior ORIF (bilat Stoppa) + posterior pelvis fixation	No	No	No	No	No	No	No	No
T15	34	M	Open pelvic Fx LC2	Crush	Direct	36		1	R SI screw	Yes	No	No	No	No	No	No	No
T16	18	M	LC2	MVA	Transfer	7		1	ORIF	No	No	No	No	No	No	No	No
F1	55	M	Open pelvic Fx LC3	Crush	Direct	14	14		LPEF + R + L SI screws	Yes	No	No	No	No	No	No	No
						median 16 (IQR 10–31)	*median 48 (IQR 37-64)	median 2 (IQR 1–3.5)									

* *Descriptive statistics for group of definitive LPEF excludes the patient who died on day 14*.

*Y-B, Young and Burgess; R, right; L, left; Fx, fracture; MVA, motor-vehicle accident; MBA, motorbike accident; SI, sacro-iliac; bilat, bilateral; IQR (interquartile range)*.

The pelvic injuries in these patients were classified, according to the Young and Burgess (11, 12) classification based on mechanism of injury, as: 1 LC1, 5 LC2, 13 LC3, 4 APC2, 1 APC3, and 3 VS. Three patients had combined pelvic and acetabular fractures. Two patients had open fractures: one patient sustained an open LC2 fracture, and the patient who died on the 14th day post-injury had an open LC3 pelvic fracture.

The most common cause of injury involved traffic accidents (18/27 patients): motorcycle accidents (seven patients), motor vehicle accidents (five patients), and pedestrian struck by motor vehicles (six patients). The other causes of injury included crush injuries (five patients), fall from a horse (two patients), and fall from own height (two patients). Six patients were transferred from peripheral hospitals. All except for one patient received the external fixator within 24 h of the injury. The median duration of stay in the hospital was 16 days (IQR 10–31). Nine patients underwent a laparotomy and one patient a thoracotomy. One patient also required a C-clamp (patient T3 with VS pattern combined with right acetabular fracture pattern). One patient received a combination of an LPEF pin and a supra-acetabular pin on the contralateral side because of the particular open fracture configuration (patient F1 with open pelvic fracture LC3).

In the patients for whom the external fixators were temporary, this was converted using various combinations of different surgical fixation methods, including anterior pelvic plating, sacroiliac screws, and posterior pelvic fixation (posterior superior iliac spine entry-point of pedicle-screw fixation system). In the short-term “definitive” treatment group, eight patients were treated with an external fixator in isolation, whereas one patient also received bilateral sacroiliac screw fixation, and one patient received posterior pelvic fixation. The patient who died at 14 days post-injury also required bilateral sacroiliac screw fixation.

In the temporary treatment group, no patients had any complications from this technique. In the short-term “definitive” treatment group, two patients sustained superficial pin site infection (cellulitis and purulent discharge), which resulted in the removal of the external fixator and washout of the wounds at 5 weeks (36 days) and 7 weeks (50 days) after initial application. One other patient had hypergranulating pin sites and a loose external fixator; however, it had functioned well and was removed at 10 weeks (71 days) after application. All the fractures obtained clinical and radiological union in good alignment.

## Discussion

We have described the use of the LPEF acutely in 27 patients with pelvic ring injuries. Numerous studies have documented beneficial results using emergent pelvic external fixation in the resuscitation phase of hemodynamically unstable polytrauma patients with unstable pelvic ring injuries (1–5). However, many different trauma resuscitation protocols exist (13). As ~80% of cases of hemodynamic instability are related to venous bleeding or fracture site bleeding (14), it is crucial to reduce the volume of the pelvis emergently to facilitate clot formation and stop ongoing venous bleeding. Indeed, it has been demonstrated in experimental studies that reduction of open-book pelvic disruptions leads to increased retroperitoneal pressures, and this is believed to contribute to a tamponade effect of venous bleeding (15). This may be achieved by using, for example, a pelvic binder, sheet or an external fixation device (including C-clamp). Pelvic packing is another method of creating a tamponade effect to control bleeding, and this may be done in the retroperitoneal space (16). Angioembolization (17) is useful to control arterial bleeding if a blush is visible on a contrast CT scan or in the case of persistent instability after pelvic binder or external fixation application. However, several trauma resuscitation protocols exist, many of which involve pelvic external fixation at some stage. Two different protocols have recently been compared in one study including 348 cases, and there was no difference in mortality, revealing that individualized trauma resuscitation protocols optimizing hospital-available resources are key in treating severely polytraumatized patients (18).

There are currently three recognized sites for pin placement in anterior pelvic external fixation: (1) anterosuperior with pins inserted perpendicular to the iliac crest, (2) supra-acetabular (or anteroinferior) with pins placed from the anterior inferior iliac spine in an anteroposterior direction, and (3) subcristal with pins placed in the anterior superior iliac spine in a direction parallel with the crest (19) (Figure 1). Solomon et al. (8) developed the subcristal approach in response to complications of pin placement in patients referred by peripheral hospitals to their trauma center. Most complications involved failure to correctly place the pin between the inner and outer tables of the ilium, injury to anatomic structures located between the skin and bony entry point, such as the lateral femoral cutaneous nerve, penetration in the hip joint, loss of fixation, and infection. Pin site infection with anterosuperior and supra-acetabular techniques have been reported as high as 20–40 and 25%, respectively, in definitive treatment cases (9, 10, 20), and Solomon et al. (8) reported a 20% incidence of superficial infection in a cohort of 20 patients with subcristal external fixators. Although the entry point of the subcristal technique, the anterior superior iliac spine, is near the lateral femoral cutaneous nerve (21), there were no cases of lateral femoral cutaneous nerve injury in the Solomon et al. (8) series.

The results from the retrospective review of our use of this technique suggest that it may be an alternative to other techniques described; further comparative research of the different techniques would be useful in the future. This technique is quick, and the pins and frame are positioned far from the abdomen, and laparotomy is easily performed in the trauma setting. Our patient cohort is quite heterogeneous, with patients of a wide age range, various pelvic disruption configurations, and various energy levels of the mechanism of injury, and our results suggest that this technique can be used in a very large range of clinical situations. One of the benefits we have found with this technique is the increased distance from the groin, and minimal movement of the skin folds around the pin sites during sitting and hip flexion as demonstrated in Figure 3, in contrast to other techniques. In fact, the two cases of pin site infections in this cohort were at 5 and 7 weeks after LPEF application, at which point the fractures had consolidated enough that the external fixators could be removed safely without any further impact on treatment.

A benefit of the LPEF technique, compared with the subcristal approach, is its ability to remain far away from the surgical field if a Pfennansteil incision were to be required later for anterior pelvic fixation (Figure 2). We believe that we have some added control of the posterior hemipelvis with the LPEF technique compared with the subcristal and iliac wing techniques due to the posterior orientation of the pins. We have also used these lateral posterior pins as temporary indirect reduction aids while undertaking definitive fixation of pelvic fractures. These pins can be used as “joysticks” to control the hemipelvis while reducing anterior pelvic fractures or pubic symphysis disruptions, as well as during percutaneous sacroiliac fixation. It can also be used in an “open” fashion if a lateral window approach is used, for instance, during open sacroiliac reduction.

One patient in the current study received a combination of a lateral posterior pin and a supra-acetabular pin on the contralateral side because of the particular fracture configuration. This is a very good case example demonstrating that several pin entry sites should be mastered by the orthopedic surgeon treating pelvic fractures, as their presentations are quite variable, and one particular technique does not necessarily fit every fracture pattern.

Our study has inherent limitations. As it is a retrospective review, there may be missing data regarding particular complications. However, we have followed up with all the patients in the present study at least until clinical and radiological union or death; therefore, we believe that we have limited the number of omissions in this study. Moreover, the number of patients in the study is limited, particularly in the definitive treatment group, as this study was done to document the initial results and describe the surgical technique of this alternative surgical technique. Furthermore, we have not collected body mass index data for this group of patients, which could influence the risk of infection and ease of surgical technique. Finally, there was no control group. Further studies with a higher number of cases and comparative groups would also be beneficial.

Our results indicate that the lateral posterior pelvic external fixator technique has a low complication rate, at least comparable with previously described techniques, likely explained by the safe anatomical profile of its insertion, inviting further comparative studies. We believe that this technique is very useful in the arsenal of techniques available to the orthopedic surgeon in treating pelvic fractures.

## Data Availability Statement

The original contributions presented in the study are included in the article/supplementary materials, further inquiries can be directed to the corresponding author/s.

## Ethics Statement

The studies involving human participants were reviewed and approved by Alfred Hospital Ethics Committee (466/13). Written informed consent for participation was not required for this study in accordance with the national legislation and the institutional requirements. Written informed consent was obtained from the individual(s) for the publication of any potentially identifiable images or data included in this article.

## Author Contributions

MR contributed to study design, performed surgeries, and reviewed the manuscript. PN and JS contributed to study design, ethics application, data analysis, and writing of the manuscript. All authors contributed to the article and approved the submitted version.

## Conflict of Interest

PN reports payment for development of educational presentations from DePuy Synthes and travel/accommodations/meeting expenses from Stryker, both unrelated to this article. The remaining authors declare that the research was conducted in the absence of any commercial or financial relationships that could be construed as a potential conflict of interest.
